# 

**Published:** 1919-03-15

**Authors:** 


### TUBERCULOSIS TOPICS.

The Central German Anti-Tuberculosis Committee has for some years, indeed before the war, had a special sub-committee dealing specially with lupus. In the *Internationales Centralblatt für Tuberkulose-Forschung*, November number, 1918, we have an abstract of the yearly report of this "lupus commission." In 1917 253 patients were treated, as against 192 in 1915, and 157 in 1916. The 253 were made up of 40 men, 164 women and 49 children. The cost of treatments seems to have been divided, the commission itself paying about a third, while the rest was borne by various bodies such as sick club, union, insurance institution, "welfare" institute, or by the patients' relations. The clinical results were 100 cured, 139 improved, 13 uncured, 1 death: thus more than a third were discharged well, and in the majority working capacity was restored. The length of treatment varied from two to five months. In many patients the treatment was ambulatory. Special provision was made for quick treatment of relapsing cases. The commission requests early information of any untreated case of lupus.

Our contemporary the *Lancet,* noticing some rather ambitious proposals for autonomy passed at a recent meeting of tuberculosis officers, has dubbed that functionary a "clinical tyro." It is to be feared that this is often the case, even as regards some whose names seem to appear rather frequently in lay newspapers; one of these, by the way, was charged recently in our contemporary's columns with plagiarism, and so far has made no answer. But when the *Lancet* goes on to say that the present system of appointment by local bodies of tuberculosis officers has no chance of being superseded we can only hope that it is wrong. Is not Professor Osler strongly in favour of a centrally administered plan and centrally elected officers? Truth to tell, the reason that so many tuberculosis officers are "clinical tyros" is just the vagaries of local election committees, who proceed very seldom on the plan of letting the best man win. The provincial, or for the matter of that, the metropolitan, councillor is all very well as regards trams, or lighting, or paving, but really he is not half, or a quarter, well educated enough to have to do with scientific matters, while, as already hinted, his eye is none too single either.

In addition to this, local jealousies make co-operation and consequent economical working impossible. We know at this minute of one inefficient little unit that has had five tuberculosis officers in four years. Another public sanatorium (belonging to a big provincial city to which the war must have brought millions upon millions sterling) had actually in this time of stress and of rampant tuberculosis empty beds, because it had to raise charges so high that the municipal authority would not pay them. Yet this sanatorium had been well established long before the rainy day of the war came; only it obstinately refused the neighbouring county's proposal to join in with it in its tuberculosis scheme. If this had been done a substantial financial basis had been obtained and stability against war prices and suchlike trials secured. This case was indeed privately investigated by the Local Government Board. We think that their visiting officials could give our contemporary many reasons for changing its views as to the desirability of local control of tuberculosis; and for coming round to the opinion of Professor Osler, which has also consistently been our own.

Now if a tuberculosis section of the Ministry of Health were formed, with at its head not mere general sanitarians, and not pushful young men either, who have for three or four years been styling themselves tuberculosis specialists—but on the contrary real phthisicologists with something good in the way of achievement—men like Arthur Latham, Clive Rivière, Egbert Morland, Gauvain of Alton—then there would be a real head for the working of the machine. Any really meritorious performance by a tuberculosis officer—this would probably not include talking—should be recognised by the offer of promotion to this body. But the system of skilled staff work once assured all the rest would follow. The wiseacres of Little Peddlington, babbling of "tubercu-lossis" and of "venereal," would be restored to their proper subjects of debate.

### The Knights Hospitallers of Rhodes.

The future of the building of the Knights Hospitallers in the Island of Rhodes, in view of its historical and archæological interests, was the subject of a question put by Mr. Bennett to the Secretary for Foreign Affairs. It was asked if an endeavour could be made to secure an undertaking from the State, to whom the island is to be assigned, that these buildings shall be kept in a state of preservation. Mr. Harmsworth said that the point would be borne in mind by the British delegates at the Peace Conference.

### The Chaplain Unnecessary?

APPARENTLY the Dunmow Guardians consider that the clergy as such have no part in poor-law administration, for it is stated that they have abolished the office of chaplain in their workhouse. It is difficult to fathom the reason for the Board's decision. Do they think a workhouse inmate is past spiritual ministration? Or do they consider a chaplain is more ornamental than useful? Or is it that they intend in future to rely upon voluntary local help for the performance of ministerial duties in their institution?

### Forthcoming Events.

The Royal Sanitary Institute has arranged Conferences on "Post-War Developments relating to Public Health," to be held on March 13, 14, and 15, at which the following subjects will be brought forward for discussion: City Hygiene in relation to Employment; Housing for City Clerks and Similar Workers; Public Health Aspect of Tuberculosis; Public Health Work and Propaganda; Welfare Work in Factories; Child Welfare Work.

Summer Congress of Laryngology.—The Section of Laryngology of the Royal Society of Medicine is showing increased energy, with the return of many of its members from the war. It has decided to convert its annual gathering in May into something more than the usual clinical meeting. On Friday, May 2, there will therefore be a Summer Congress. Papers will be read in the morning; demonstrations of cases, operations, specimens, instruments will take place in the afternoon; and it is proposed to arrange a pathological museum of specimens relating to the subject. All oversea laryngologists will be heartily welcomed. Those who intend to read papers or join in the discussion are requested to notify the Hon. Secretaries not later than March 3.

The Child-study Society, London.—Lectures and discussions will be held at the Royal Sanitary Institute, 90 Buckingham Palace Road, S.W. 1, on the following named dates at 6 p.m.:—March 20, Discussion on "Training of the School Girl in Infant Care," Mrs. K. Truelove, opener; chairman, F. Truby King, C.M.G., M.B., B.Sc. April 3, "Home *v.* Institutional Training of Young Children," by Eric Pritchard, M.A., M.D., M.R.C.P.; chairman, the Hon. Sir John A. Cockburn, K.C.M.G., M.D.

St. Thomas's Hospital Medical School.—In connection with the University of London a series of ten lectures will be given in the Governor's Hall of the hospital on "Diseases Met with in the Sub-tropical War Areas." These lectures will be given at 5 p.m. on Wednesdays and Fridays, and commenced on March 5, the lecturer being Leonard S. Dudgeon, C.M.G., F.R.C.P. (Lond.), late Temp. Colonel A.M.S. and consulting bacteriologist to the British Forces in the Balkans and member of the War Office Committee for Infectious Diseases, Eastern Mediterranean. The lectures are open to students of London University and all medical practitioners. Medical officers of the Allied Forces are specially invited.

The Home Hospitals Association for Paying Patients.—The forty-first ordinary general meeting will be held at Fitzroy House, Fitzroy Square, London, W., on Friday, March 21, 1919, at 5 p.m., Sir John Bland Sutton, F.R.C.S., in the chair.

Poplar Hospital for Accidents.—Annual General Meeting at the hospital Tuesday, March 18, 1919, at 5.30 o'clock.

National Hospital for the Relief and Cure of the Paralysed and Epileptic.—The annual meeting of governors and members will be held at the hospital on Tuesday, March 25, at 4.30 p.m.

### PASSING EVENTS AND TOPICS.

**Making Use of Smallpox Hospital.**

New buildings for a smallpox hospital at Cheshunt are now practically completed, and as there are no smallpox patients, the County Council proposes to use the place for acute and advanced cases of tuberculosis.

**The Hay Box in the Hospital.**

Although some latitude is allowed the hospitals in the matter of fuel rationing, it is up to every management to exercise the greatest economy in every possible direction, and it is therefore interesting in this connection to learn that in one London institution sufficient porridge for forty patients is daily prepared in a hay box which the matron has now introduced to save gas and coal in the kitchen department.

**Home for Blind Babies.**

The National Institute for the Blind have recently inaugurated an institution which is in itself unique, and takes the form of a Home for Blind Babies. The Home, which is known as "Sunshine House," stands in its own grounds of nine acres, is situated at Chorley Wood, Herts, and is capable of accommodating some twenty-five babies, who are under the care of a matron and staff. The building is suitably equipped with miniature furniture, pictures in dormitories, and brightly coloured china; the whole idea being to bring up the little ones normally. The present plan is to retain the babies until five years of age, then passing them on to other institutions for special training.

---

When the Ministry of Munitions disappears the welfare work in factories will, it is officially announced, continue under the Ministry of Labour and be enlarged in scope.

Acting on a recommendation of the Medical Officer of Health that incubators should be used for rearing prematurely born babies, the Ilford Urban District Council is seeking sanction from the Local Government Board to incur the necessary expense.

The Chelsea Hospital for Women has received from Colonel Charles Gulliver a cheque for £277 11s. 9d., being the proceeds of a Matinee held at the Palladium Theatre on January 3 in aid of the hospital.

### Matrons' and Sisters' Appointments.

City Hospital Annexe, Fazakerley, Liverpool.—Miss Fanny Forster has been appointed sister. She was trained at Toxteth Infirmary, Liverpool, and has been night sister at the Northern Hospital, Manchester.

Frimley District Cottage Hospital.—Miss Mary E. Leng has been appointed nurse-matron. She was trained at the North Riding Infirmary, Middlesbrough; has been matron of the Ulverston Cottage Hospital, the Penrith Cottage Hospital, Badley Nurses' Home, Dudley; holiday matron at the Wingfield Hospital, Oxford; acting sister at Policlinico, Rome; and sister at the 3rd London Hospital, Oxford.

Royal Hospital for Sick Children, Aberdeen.—Miss Anna M. Carter and Miss Jean M. Steel have been appointed surgical sister and night sister respectively. They were both trained at Dumfries and Galloway Royal Infirmary, where they served later as sister and temporary night sister. Miss Carter has been matron of Broomlands Auxiliary Hospital, Dumfries, and Miss Steel has been nursing sister at the Craigleith Military Hospital, Edinburgh, and matron of the Castle Mill Auxiliary Hospital, Lockerbie.

Victoria Hospital, Swindon.—Miss Mary Warral has been appointed sister. She was trained at the Hereford County Hospital, and has been staff nurse and sister at the General Hospital, Loughborough.

Isolation Hospital, Derby.—Miss Elsie Maud Lea has been appointed ward sister. She was trained at the Infirmary, Rochdale, and at the Borough Isolation Hospital, Derby.

Children's Hospital, Sheffield.—Miss Edith E. Bembridge has been appointed ward sister. She was trained at the Royal Hospital, Sheffield, and at the City Hospital, Seacroft, Leeds.

General Hospital, Walthamstow.—Miss Margaret Robertson has been appointed sister. Trained at Guy's Hospital, she has been on the private nursing staff of that institution, and has engaged in private nursing at Eastbourne. She holds the C.M.B. certificate.

Royal National Hospital, Ventnor.—Miss Margaret A. H. Fairbairn has been appointed night sister. Trained at the Royal Victoria Hospital, Newcastle-on-Tyne, Miss Fairbairn has held appointments in Carlisle and Wrexham.

Hospital for Infectious Diseases, Bournemouth.—Miss Eva Wilcox has been appointed matron. Trained at Fulham Infirmary and Parkhill Hospital, Liverpool, Miss Wilcox has been night superintendent, City Hospital, Newcastle-on-Tyne; tuberculosis health visitor, Halifax; sister of a tuberculosis sanatorium in France; matron of the Weston Road Hospital, Runcorn; matron, Infectious Diseases Hospital and Tuberculosis Sanatorium, Yarnfield; and has served with the Scottish Women's Hospitals in Russia, Roumania, and France. She was awarded the Military Medal of St. George of Russia in 1917.

Kensington and Fulham General Hospital, Earl's Court.—Miss Lorna B. Wood, R.R.C., has been appointed matron. She was trained at Guy's Hospital, where she was later sister-housekeeper, and also a member of the private nursing staff. She has been matron of the Red Cross Hospital, Montezah, Egypt, has done general district nursing at Southwark, and has held a post under Queen Alexandra's Royal Naval Nursing Service.

Queen Alexandra's Military Nursing Service for India.—Miss Dorothy Webley has been appointed nursing sister.

The Infirmary, Kilmarnock.—Miss L. Kavanagh has been appointed surgical sister. She was trained at the Fulham Infirmary, Hammersmith, later being staff nurse there; has been temporary sister at the Abram Peel War Hospital, Bradford, and night sister and ward sister under the British Red Cross Society.

Clare Hall Hospital, South Mimms.—Miss Gladys Stocking has been appointed night sister. She was trained at St. Mary's Hospital, Paddington, has done military nursing at the No. 8 General Hospital, France, and the War Hospital, Huddersfield, and has been temporary staff charge nurse at the Children's Hospital, Kensington.

Springfield Hospital, Rochdale.—Miss Annie Robinson has been appointed night sister. She was trained at the North Bierley Infirmary, Clayton, Bradford, where she has since been staff nurse and sister.

Rochdale Municipal Maternity and Infants' Hospital.—Miss Frances Bambridge has been appointed sister. She was trained at the Hackney Infirmary, Homerton, and has been nursing in Siberia, Russia, and at the Stoke-on-Trent War Hospital.

Alexandra Hospital, Montreal, Canada.—Miss Martha Cutforth has been appointed lady superintendent. She was trained at St. Leonard's Infirmary, Shoreditch, and has since been ward sister at Kingston Infirmary and Stroud General Hospital, and assistant superintendent at Alexandra Hospital, Montreal. She has also done private nursing in London and Brussels.

Cottage Hospital, Llandudno.—Miss Brown has been appointed matron. She was trained at the London Hospital, and has been matron of the Urmston Cottage Hospital.

The Infirmary, Harrogate.—Miss Minnie Willis has been appointed sister. She was trained at Durham County Hospital, where she was later night and day sister.

### National Baby Week Competitions.

The National Baby Week Council announces the following competitions for this year:

Open Competitions.—Prizes offered for—(1) A week's programme of how to make an infant-welfare centre attractive and instructive—(a) in town; (b) in the country. (2) The three best original postcards in black and white. (3) The best set of bold original illustrations for decoration of infant-welfare centre. (4) The best motto, with pictorial representation, suitable for Baby Week. (5) The best original toy, the parts so constructed that it can be dissected and rebuilt by a child.

Special Competitions.—(1) For school teachers only: The best suggestions, worked out with examples, for a School Baby Week (remembering that 1919 is an Imperial Week)—(a) in town, or (b) in the country. (2) For infant-school teachers only: The best scheme for short and easy "Health Chats" for tiny children, the gist of which might be "taken home to mother." (3) For girls under eighteen only: The best set of miniature baby clothes for a doll (consideration given to using up "odd bits"). (4) For boys under eighteen only: The best home-made toy (washable).

All articles and essays sent in for the Competition will be the property of the Council, and must reach the office not later than June 1. Full particulars as to regulations and prizes may be obtained from the Secretary, 27a Cavendish Square, London, W. 1.

### Gifts and Bequests.

Miss A. E. Arkwright, of Portman Square and Algiers, left £500 to the British Cottage Hospital at Algiers, and £200 to All Saints' Church, Algiers, on the death of her brother.

Mr. and Mrs. Leonard Firth, of Leeds, have given £1,000 to the Dewsbury and District Infirmary to endow a bed in memory of their son, Second Lieutenant Philip Firth, who was killed in a flying accident in Yorkshire last October.

Mr. Francis James Fry, chocolate manufacturer, left a fortune of the value of £500,000 "so far as at present can be ascertained." Mr. Fry bequeathed a sum not exceeding £4,000 to the executors for distribution amongst charitable objects in which he was interested, including, if thought fit, the Royal Infirmary, the General Hospital and Dispensary, Bristol.

Miss Ellen Mary Gibson, of Oxton, Cheshire, bequeathed £200 to Leasowe Hospital for Crippled Children, and £250 to the Queen's Hospital, Birmingham.

Colonel Robert Watson Sparks, of Richmond, bebequeathed £100 each to the Royal Hospital, Richmond, the Westminster Hospital, the Richmond Association of the London City Mission, and Miss Daniell's Soldiers' Home, Aldershot.

Mrs. A. M. Phelps, of Cheltenham, left £1,000 to the Cheltenham General Hospital and £300 each to the Delancy Fever Hospital and the Eye, Ear and Throat Hospital at Cheltenham.

Miss Maud Chatfield, of Southport, left £500 each to the Liverpool Country Hospital for Children (Southport), The Croydon General Hospital, and four Southport Medical charities, and £500 to the London Hospital.

